# Clinical characteristics and prognosis of breast cancer patients with ovarian metastases

**DOI:** 10.3389/fonc.2025.1640067

**Published:** 2025-09-23

**Authors:** Chunyan Gao, Yan Li, Xiaoping Ma, Zhenhui Zhao, Li Li, Dan Liu, Bingyu Li, Bing Zhao

**Affiliations:** Department of Breast Cancer, Affiliated Tumor Hospital of Xinjiang Medical University, The Clinical Research Center of Breast Tumor and Thyroid Tumor in Xinjiang Uygur Autonomous Region, Urumqi, Xinjiang, China

**Keywords:** breast cancer, ovarian metastasis, invasive lobular carcinoma, invasive ductal carcinoma, OS after OM

## Abstract

**Objective:**

This study aims to investigate the clinical characteristics, overall survival (OS), and prognostic factors associated with breast cancer patients who have ovarian metastasis (OM).

**Materials & methods:**

This retrospective single-center study analyzed 41 breast cancer patients with pathologically confirmed OM who underwent bilateral oophorectomy between 2011 and 2022. Data on clinical-pathological characteristics, molecular subtypes, detection methods, and survival were collected. The survival times were analyzed using Kaplan-Meier survival analysis. Prognostic factors were assessed through Cox regression models.

**Results:**

The cohort consisted of 33 (80.5%) invasive ductal carcinoma (IDC) and 8 (19.5%) invasive lobular carcinoma (ILC) cases. Most patients (87.8%) were hormone receptor-positive. Patients with ILC were significantly older at initial diagnosis than IDC patients (median 45 *vs*. 37 years, P = 0.014). OM was detected earlier in IDC, often incidentally during ablation surgery (54.5% *vs*. 12.5% for ILC, P = 0.032), whereas ILC patients typically presented with symptoms. For the entire cohort, the median OS was 85.0 months, while the median OS after OM was 28 months. Notably, patients with ILC exhibited a significantly shorter OS after OM (11.5 months) compared to those with IDC (30 months; P = 0.01). Furthermore, the interval from the initial diagnosis of breast cancer to the emergence of OM was significantly associated with the OS of these patients (P < 0.05), serving as an independent prognostic indicator.

**Conclusions:**

OM, which may not exhibit overt clinical manifestations in the early stage, significantly affects the survival of BC patients. The ILC histological type is associated with a particularly unfavorable post-OM prognosis, and the interval from initial diagnosis to OM is a key prognostic indicator. These findings may guide clinical management in these patients.

## Introduction

According to the most recent global statistics on malignant tumors from 2020, breast cancer (BC) has surpassed lung cancer as the most prevalent malignant tumor among females, with approximately 2.3 million new cases (11.7%) diagnosed annually. Although the mortality rate for BC ranks fourth among malignant tumors, it remains the leading cause of cancer-related deaths in female patients. This is particularly evident in developing countries, where the BC mortality rate is as high as 15 per 100,000, significantly higher than that in developed countries at 12.8 per 100,000 (1). Metastatic BC is a critical factor contributing to the high BC mortality rate, with a median overall survival (OS) of only 2 to 3 years for affected patients. Clinically, approximately 25% to 28% of patients present with advanced disease at diagnosis, while recurrent or metastatic BC accounts for about 20% to 30% of cases. The most common metastatic sites for BC include the bones, lymph nodes, liver, lungs, brain, and adrenal glands (2, 3). Although a small proportion of BC cases metastasize to the ovaries, BC remains one of the primary sources of secondary malignant tumors in the ovaries (4). There is a higher incidence of secondary ovarian malignancies in Asian countries than in Western countries, which may be associated with the increased incidence of primary tumors (5).

Different molecular subtypes of BC exhibit different characteristics, and tumor size is significantly associated with human epidermal growth factor receptor 2 (HER2) status (6). The sites of metastasis in BC, particularly the initial metastatic site, show significant correlations with molecular subtypes of BC. Hormone receptor-positive BC is more likely to develop bone metastasis, whereas HER2-positive BC predominantly metastasizes to the liver and lungs. Additionally, liver and brain metastases are more prevalent in basal-like BC (7). Studies have indicated that ovarian metastasis (OM) primarily occurs in invasive lobular carcinoma (ILC) and hormone receptor-positive BC (8–10), often accompanied by additional metastatic sites. The prognosis for BC patients with OM is unfavorable, with a 5-year survival rate ranging from 6% to 26% (11). The average survival time for BC patients with OM is 25 months (12). Early detection of BC metastasis is widely recognized as an effective strategy for managing disease progression and prolonging OS (13). Therefore, both common and rare metastatic sites of BC should receive sufficient attention.

Although published reports on BC with OM exist, the field remains limited by studies with relatively small sample sizes, often comprising fewer than 30 cases (11, 12, 14, 15). Furthermore, many previous investigations have focused on isolated aspects, such as the association with molecular subtypes or patient age (8, 16), rather than providing a comprehensive analysis that integrates histopathological type, clinical presentation, and prognostic outcomes. This study, which constitutes one of the largest single-center cohorts of pathologically confirmed OM, aims to address these gaps by providing a detailed comparison of invasive ductal carcinoma (IDC) and ILC in the context of OM, and by analyzing factors influencing OS and survival after OM.

## Materials and methods

### Study participants

This retrospective study analyzed the clinical data of 41 BC patients with OM who underwent bilateral oophorectomy at the Affiliated Tumor Hospital of Xinjiang Medical University between April 2011 and February 2022. The primary tumors and OMs of all patients were pathologically confirmed. The pathological characteristics collected included histological type, distant metastasis, lymph node involvement, estrogen receptor (ER) status, progesterone receptor (PR) status, HER2 status, and Ki-67 index. Clinical characteristics, including age at initial diagnosis, age at OM diagnosis, duration from initial diagnosis of primary tumor to OM, first metastatic site, methods of OM detection, presence of peritoneal metastases or peritoneal lymph node involvement, ascites, and levels of CA-125 and CA-15-3, as well as OS and OS after OM, were also documented.

The inclusion and exclusion criteria were as follows: 1) All cases of BC were confirmed as invasive carcinoma through either biopsy or surgical pathology at initial diagnosis; 2) Patients with complete clinical and pathological data; 3) OM was confirmed through pathological evaluation following bilateral oophorectomy, and the diagnosis complied with the pathological and immunohistochemical characteristics of BC metastasis. 4) Patients with other concurrent or metachronous primary malignant tumors were excluded from the study; 5) Patients received personalized antitumor treatment based on their clinical staging and molecular subtyping after the initial diagnosis of BC. This treatment included chemotherapy, radiotherapy, endocrine therapy, and targeted therapies, with specific protocols determined by clinical practice and current treatment guidelines. 6) There were no standardized restrictions on treatment protocols following the diagnosis of OM. The treatment administered was based on actual clinical records, and complete follow-up data were available.

Molecular typing of BC was performed based on the consensus of the 2015 St. Gallen International BC Conference (17), using the immunohistochemical method. The Luminal A subtype was defined by positive ER status, PR status of 20% or greater, a Ki-67 index of less than 20%, and negative HER2 status. Luminal B type included two categories: Subtype 1, characterized by negative HER2 status, positive ER status, PR less than 20%, or a Ki-67 index of 20% or greater; and Subtype 2, defined as HER2-positive, ER-positive, and any level of PR and Ki-67 index. The HER2-overexpressing subtype included negative ER and PR statuses with positive HER2 status. The triple-negative subtype was defined by negative ER, PR, and HER2 statuses. The TNM staging utilized adhered to the 8th edition of the American Joint Committee on Cancer (AJCC) breast cancer staging system (18).

### Follow-up

Follow-up assessments were conducted through inpatient visits, outpatient appointments, and telephone consultations to monitor patient survival, tumor recurrence, and metastasis. The follow-up period extended from the date of pathological diagnosis of BC to the date of the patient’s death due to cancer or the end of the last follow-up. The follow-up ended in June 2022. The OS was defined as the duration from the date of BC diagnosis to the date of death. OS after OM referred to the time from the date of bilateral oophorectomy to the date of death.

### Statistical methods

Statistical analyses were performed using SPSS version 25.0. Descriptive statistics were used to summarize the general conditions and clinical characteristics of the patients. The chi-squared (χ²) test was applied to compare differences among various histological types. The median survival time was analyzed using the Kaplan-Meier method, and the 95% confidence interval (CI) was calculated. The differences in survival were compared using the log-rank test. Survival analysis and visualization were performed using R packages. Univariate and multivariate Cox regression analyses were conducted to identify factors influencing OS and OS after OM. A p-value of less than 0.05 was considered statistically significant.

## Results

### General clinical features

A total of 41 BC cases with OM were included in this study. The age at initial diagnosis of BC ranged from 29 to 59 years, with a median age of 39 years. The interval between initial diagnosis and the occurrence of OM varied from 6 to 192 months, and the median interval was 38 months. Among the patients, 33 (80.5%) had IDC, while 8 (19.5%) were diagnosed with ILC. At initial diagnosis, only one case (2.4%) was classified as stage I and identified as triple-negative. A total of 37 cases (90.2%) were classified as stage I-III, while 4 cases (9.8%) were classified as stage IV at initial diagnosis. Furthermore, 18 (43.9%) had left-sided BC, and 23 (56.1%) had right-sided BC. In terms of molecular typing, 36 cases (87.8%) were ER positive or PR positive; 2 cases (4.9%) were HER2 positive; 1 (2.4%) case was classified as Luminal A; 35 (85.4%) cases were Luminal B; 2 cases (4.9%) were HER2-overexpressing; and 3 cases (7.3%) were classified as triple-negative.

Among the 41 patients, 19 (46.3%) were diagnosed following ovarian ablation surgery, while 22 (53.7%) were diagnosed after surgical resection due to clinical symptoms, including abdominal pain, vaginal bleeding, abdominal mass, or abnormal imaging findings on gynecological ultrasound, pelvic CT, or pelvic MRI. Twenty-six patients (63.4%) presented with bilateral OM, while 15 patients (36.6%) had unilateral OM. All patients underwent bilateral oophorectomy. Additionally, 15 patients (36.6%) had OM accompanied by peritoneal lesions (such as peritoneal metastasis, retroperitoneal lymph node metastasis, or ascites), while the remaining 26 patients (63.4%) did not have peritoneal lesions. **Table 1** provides an overall view of the characteristics of the 41 patients.

**Table 1:** Characteristics of 41 BC patients with OM.

Variables	Characteristics	N	Proportion (%)
Age of onset (years)	≤35	15	36.6
	>35	26	63.4
Time from BC to OM (months)	0-12	8	19.5
	13-36	12	29.3
	37-60	4	9.7
	61-192	17	41.5
Site of first metastasis	Lymph nodes/Chest wall	7	17.1
	Bone	10	24.4
	ovary	18	43.9
	viscera	6	14.6
Histological type	IDC	33	80.5
	ILC	8	19.5
ER/PR	Positive	36	87.8
	Negative	5	12.2
HER-2	Positive	2	4.9
	Negative	39	95.1
Molecular subtypes	Luminal A	1	2.4
	Luminal B	35	85.4
	HER-2 overexpression	2	4.9
	Triple negative type	3	7.3
T	1	7	17.1
	2	30	73.2
	3	2	4.9
	4	2	4.9
N	0	9	22.0
	1	10	24.4
	2	9	22.0
	3	13	31.7
M	1	4	9.8
	0	37	90.2
TNM staging of the tumor	1	1	2.4
	2	19	46.3
	3	17	41.5
	4	4	9.8
Primary BC	Right	23	56.1
	Left	18	43.9
OM	Bilateral	26	63.4
	Right	10	24.4
	Left	5	12.2
Ovarian ablation	Yes	19	46.3
	No	22	53.7
Intraperitoneal metastasis	Yes	15	36.6
	N0	26	63.4

BC, Breast Cancer; OM, Ovarian Metastasis; IDC, Invasive Ductal Carcinoma; ILC, Invasive Lobular Carcinoma; ER, Estrogen Receptor; PR, Progesterone Receptor; HER-2, Human Epidermal Growth Factor Receptor 2; T, Primary Tumor size (based on AJCC TNM staging system); N, Regional Lymph Node status (based on AJCC TNM staging system); M, Distant Metastasis status (based on AJCC TNM staging system); TNM, Tumor, Node, Metastasis (AJCC Cancer Staging System).

### Differences in pathological types of BC with OM

In the current study, the median age at initial diagnosis for IDC was 37 years, with a median interval from initial diagnosis to OM of 36 months. In contrast, for ILC, the median age at initial diagnosis was 45 years, and the median interval from initial diagnosis to OM was 66 months. The Mann-Whitney U test indicated a statistically significant difference in age at initial diagnosis between the two histological types (U = 58.00, P = 0.014). Although the median time from initial diagnosis to OM was longer for ILC than for IDC, this difference did not reach statistical significance (P > 0.05).

The age composition at initial diagnosis was compared between the IDC and ILC groups using the χ² test. In the IDC group, 45.5% of patients were aged 35 years or younger, while 55.5% were older than 35 years. In contrast, all patients in the ILC group were older than 35 years. The difference in age composition between the two groups was statistically significant (P = 0.017). Furthermore, the distribution of lesion sites regarding primary tumors (left or right) at initial diagnosis was also compared between the two groups; however, this comparison revealed no significant difference (P = 0.230). Additionally, the proportions of bilateral OM were 69.7% in the IDC group compared to 37.5% in the ILC group, while unilateral OM was observed in 30.3% and 62.5% of patients in the respective groups. The difference was not statistically significant (P = 0.09). Lastly, the detection methods for OM were compared between the two groups using the χ² test. In the IDC group, 54.5% of patients had their OM detected following ovarian ablation surgery without clinical symptoms or abnormal imaging findings, compared to 12.5% in the ILC group. Conversely, 45.5% of patients in the IDC group and 87.5% in the ILC group had their OM identified through clinical symptoms or imaging findings confirmed by postoperative pathology. The differences in detection methods were statistically significant between the two groups (P = 0.032) (**Table 2**).

**Table 2:** The difference between IDC and ILC.

Variables	Groups	IDC	ILC	χ^2^	P
Age of onset (years)	≤35	15	0	5.734	0.017
	>35	18	8		
Primary BC	Right	17	6	1.442	0.230
	Left	16	2		
OM	Bilateral	23	3	2.877	0.090
	unilateral	10	5		
Ovarian ablation	Yes	18	1	4.578	**0.032**
	No	15	7		

IDC, Invasive Ductal Carcinoma; ILC, Invasive Lobular Carcinoma; BC, Breast Cancer; OM, Ovarian Metastasis.

P = 0.017 for "Age of onset (years)" and P = 0.032 for "Ovarian ablation", respectively indicating statistically significant differences between IDC and ILC in age at onset distribution and ovarian ablation status.

### OS and OS after OM

**Figure 1:**
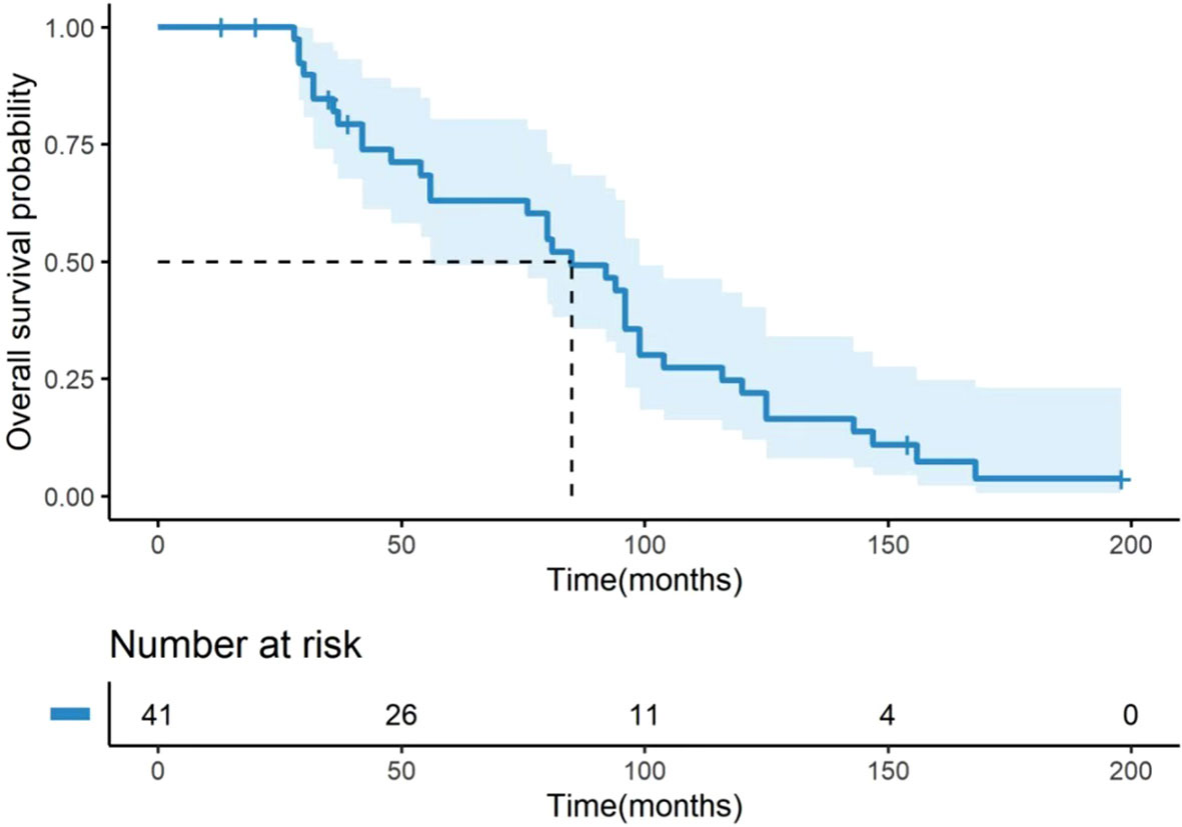
Kaplan-Meier curve for OS of 41 BC patients with OM. OS was defined as the time from the initial breast cancer diagnosis to the date of death from any cause. The median OS for the entire cohort was 85.0 months (95% CI: 67.35–102.65). The number of patients at risk at specific time points is presented below the figure. OS, overall survival; BC, breast cancer; OM, ovarian metastases; CI, confidence interval.

**Figure 2:**
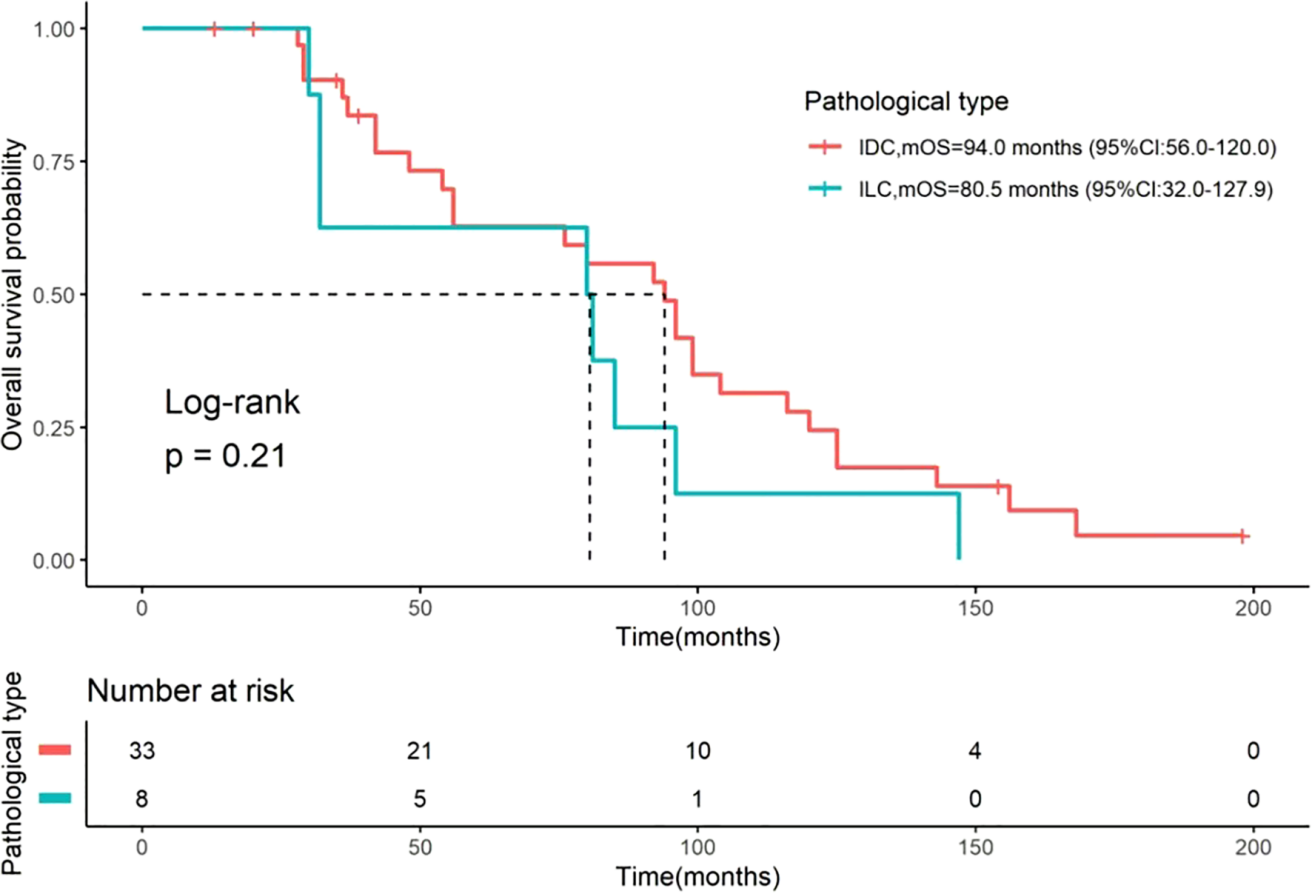
Kaplan-Meier curves for OS stratified by histological type. Comparisons of OS between patients with IDC (n=33) and ILC (n=8). The median OS was 94.0 months (95% CI: 56–120) for IDC and 80.5 months (95% CI: 32–127.9) for ILC. The difference between the two groups was not statistically significant (Log-rank test, P = 0.21). The number of patients at risk at specific time points is presented below the figure. OS, overall survival; IDC, invasive ductal carcinoma; ILC, invasive lobular carcinoma; CI, confidence interval.

**Figure 3:**
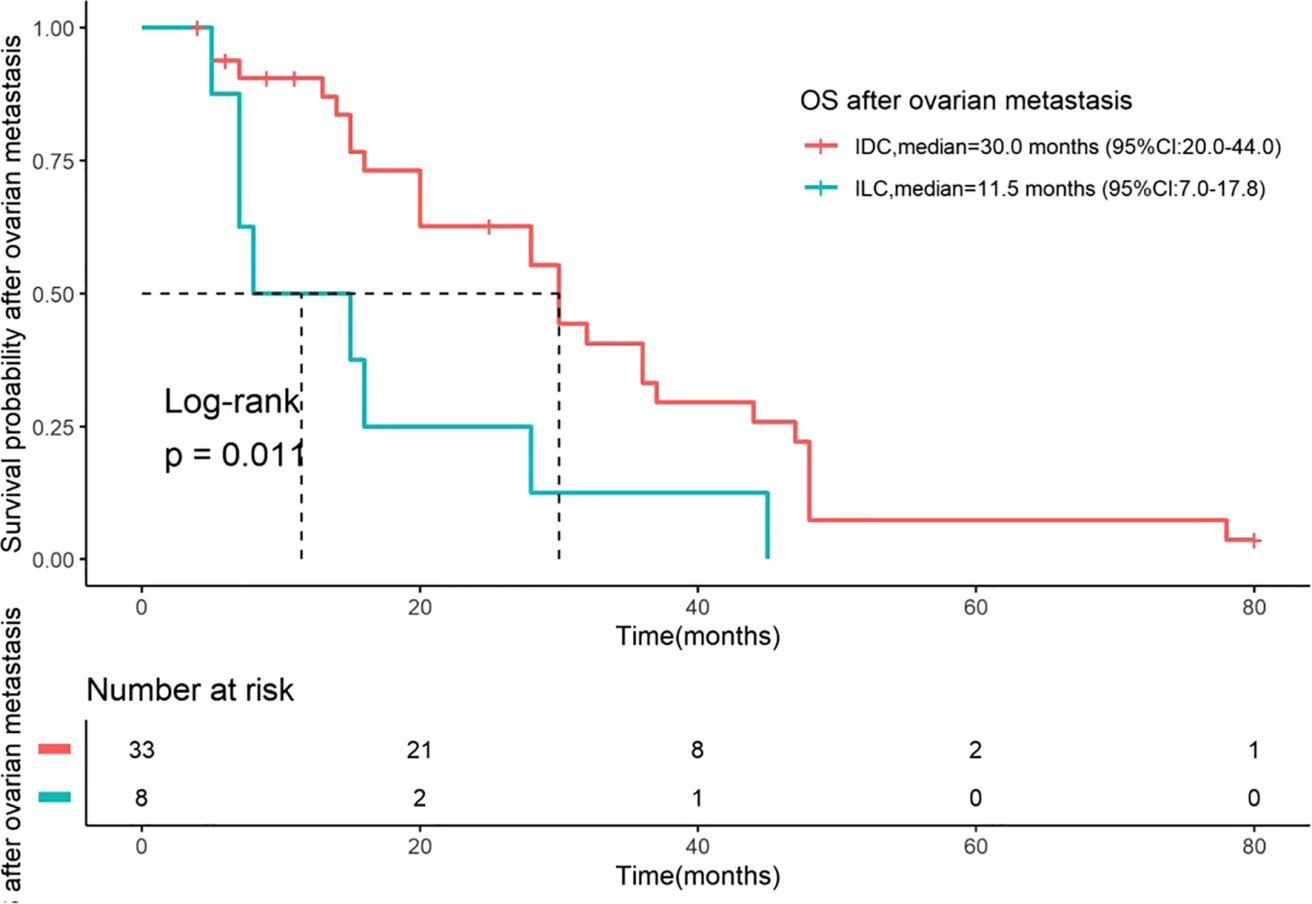
Kaplan-Meier curves for OS after OM stratified by histological type. OS after OM was defined as the time from the date of bilateral oophorectomy to the date of death. Comparisons are between patients with IDC (n=33) and ILC (n=8). The median OS after OM was 30.0 months (95% CI: 20.0–44.0) for IDC and 11.5 months (95% CI: 7.0–17.8) for ILC. The difference between the two groups was statistically significant (Log-rank test, P = 0.01). The number of patients at risk at specific time points is presented below the figure. OS, overall survival; OM, ovarian metastases; IDC, invasive ductal carcinoma; ILC, invasive lobular carcinoma.

Among the 41 patients, 35 (85.4%) died. The median OS was 85.0 months (95% CI: 67.35–102.65, **Figure 1**) for all patients, 94.0 months (95% CI: 56–120) for those with IDC, and 80.5 months (95% CI: 32–127.9) for those with ILC (**Figure 2**). The difference in OS between the IDC and ILC groups was not statistically significant (P = 0.21, **Figure 2**). The median OS after OM was 28 months (95% CI: 16.0–36.0) for all patients, 30.0 months (95% CI: 20.0–44.0) for patients with IDC, and 11.5 months (95% CI: 7.0–17.8) for those with ILC. Notably, the difference in OS after OM was statistically significant (P = 0.01) between the IDC and ILC groups (**Figure 3**). Additionally, OS after OM did not differ significantly for patients whose OM was detected through ovarian ablation surgery compared to those diagnosed by non-ablation methods (clinical symptoms and imaging findings confirmed by postoperative pathology). The median OS after OM was 30 months (95% CI: 20.3–39.7) for the former and 16 months (95% CI: 9.1–22.9) for the latter.

### Univariate analysis of factors affecting OS

A univariate Cox regression analysis was conducted on 14 factors that may affect the OS of BC patients with OM. The results indicated that the duration from the initial diagnosis of BC to OM, the first metastatic site, HER2 expression, and distant metastasis at the initial diagnosis of the primary tumor were significantly associated with patient OS (P < 0.05, **Table 3**).

**Table 3:** Cox regression analysis of OS.

Variables	Univariate analysis		Multivariate analysis	
	P	HR	P	HR (95%CI)
Age of onset (>35/≤35 years)	0.480	1.279		
Time from BC to OM	<0.001		0.005	
0-36 months (Reference)				
37-72 months	0.495	0.718	0.336	0.570 (0.181–1.794)
73-192 months	<0.001	0.202	0.001	0.157 (0.051–0.488)
OM size	0.547	1.028		
Site of first metastasis	0.070		0.061	
Lymph nodes/Chest wall (Reference)				
Bone	0.206	2.382	0.021	7.190 (1.348–38.342)
Ovary	0.010	5.051	0.042	5.017 (1.058–23.804)
Viscera	0.044	3.143	0.008	8.290 (1.732–39.677)
Histological type (ILC/IDC)	0.221	1.653		
ER/PR (Positive/Negative)	0.549	0.739		
HER-2 (Positive/Negative)	0.011	7.592	0.106	4.318 (0.734–25.391)
T	0.552			
1 (Reference)				
2	0.384	1.616		
3	0.756	1.312		
4	0.154	3.665		
N	0.912			
0 (Reference)	0.546	1.380		
1	0.651	1.261		
2	0.526	1.326		
3	0.546	1.380		
M (1/0)	0.033	3.554	0.067	15.976 (0.827–38.352)
Tumor staging	0.115		0.312	
1 (Reference)				
2	0.283	3.128	0.133	7.369 (0.545–49.567)
3	0.227	3.683	0.170	5.991 (0.465–37.131)
4	0.040	11.791	0.067	15.967 (0.827–108.352)
Primary BC (Left /Right)	0.154	1.628		
OM (Bilateral/unilateral)	0.152	1.704		
Ovarian ablation (Yes/No)	0.870	1.058		
Intraperitoneal metastasis (Yes/No)	0.529	0.803		

OS, Overall Survival; HR, Hazard Ratio; CI, Confidence Interval; BC, Breast Cancer; OM, Ovarian Metastasis; IDC, Invasive Ductal Carcinoma; ILC, Invasive Lobular Carcinoma; ER, Estrogen Receptor; PR, Progesterone Receptor; HER-2, Human Epidermal Growth Factor Receptor 2; T, Primary Tumor size (based on AJCC TNM staging system); N, Regional Lymph Node status (based on AJCC TNM staging system); M, Distant Metastasis status (based on AJCC TNM staging system).

### Multivariate analysis of factors affecting OS

The four factors with P < 0.2 from the univariate Cox regression analysis were included in the multivariate Cox regression model. As shown in **Table 3**, the duration from the initial diagnosis of BC to OM was identified as a significant independent prognostic factor for OS in BC patients with OM (P < 0.05; **Table 3**).

### Univariate analysis of factors affecting OS after OM

A univariate Cox analysis was performed on nine characteristics that might affect OS after OM. The analysis revealed that three features significantly affected OS after OM: unilateral or bilateral OM, presence of metastasis in peritoneal lymph nodes or peritoneum, or ascites, and histological type of BC at initial diagnosis (P < 0.05). Other factors, including age at OM, OM size, whether OM was identified through ovarian ablation surgery, the interval from the first diagnosis of BC to OM, and CA-15–3 levels, were not associated with OS after OM (**Table 4**).

**Table 4:** Cox regression analysis of OS after OM.

Variables	Univariate analysis		Multivariate analysis	
	P	HR	P	HR (95%CI)
Onset age (>35/≤35 years)	0.419	0.753		
OM (Bilateral/unilateral)	0.071	2.012	0.09	3.525 (1.374–9.044)
Intraperitoneal metastasis (Yes/No)	0.010	2.928	0.109	1.982 (0.859–4.574)
Size of OM	0.105	1.074		
Ovarian ablation (Yes/No)	0.262	0.677		
Histological type (ILC/IDC)	0.017	2.741	0.005	4.857 (1.632–14.459)
Time from BC to OM	0.907			
0-36 months (Reference)				
37-72 months	0.720	1.179		
73-192 months	0.709	1.158		
CA-125 (Positive/Negative)	0.052	1.975	0.351	1.443 (0.667–3.122)
CA-15-3 (Positive/Negative)	0.722	0.882		

OS, Overall Survival; OM, Ovarian Metastasis; HR, Hazard Ratio; CI, Confidence Interval; ILC, Invasive Lobular Carcinoma; IDC, Invasive Ductal Carcinoma; BC, Breast Cancer; CA-125, Cancer Antigen 125; CA-15-3, Cancer Antigen 15-3.

### Multivariate analysis of factors affecting OS after OM

The four factors with P < 0.2 from the univariate analysis were included in the multivariate Cox regression model. The analysis revealed that only the histological type of BC at initial diagnosis was significantly associated with OS after OM, serving as an independent risk factor for OS (P < 0.05; **Table 4**).

## Discussions

The most common source of ovarian secondary malignant tumors is colorectal cancer, followed by BC, which accounts for approximately 8.4% to 15% (19–21). Early observational studies based on autopsy and surgical data have demonstrated differences in metastatic sites between non-special invasive carcinoma and ILC. ILC, in addition to common metastatic sites such as the lungs, bones, liver, pleura, and brain, frequently spreads to rarer organs, including the meninges, peritoneum, gastrointestinal tract, ovaries, and adrenal glands (22–25). In the present study, we included the largest number of cases of BC with OM to date. Specifically, there were 8 cases (19.5%) with a primary tumor of ILC and 33 cases (80.5%) with a primary tumor of IDC. This distribution is consistent with previous findings indicating that the overall prevalence of ILC is lower compared to IDC among all BC cases (26–29). In contrast, the proportion of ILC in our cohort is lower than reported in previous studies, which indicates that the proportions of ILC in BC with OM range from 50% to 61% (12, 14). This discrepancy may be attributed to differences in patient populations across institutions, as our study excluded patients with OM diagnosed through biopsy or imaging examination in our study.

Molecular characterization of our cohort revealed that 36 cases (87.8%) were hormone receptor-positive (ER or PR positive), with one (2.4%) case classified as Luminal A and 35 cases (85.4%) classified as Luminal B subtype. This is consistent with previous findings that hormone receptor positivity is more common in BC patients with OM (8, 11, 12, 15, 27, 30). Interestingly, research on unilateral and bilateral OM in the context of secondary ovarian cancer and BC indicates that bilateral OM is more frequent (4, 12, 15, 19, 31, 32). Consistently, our results showed that 63.4% (26/41) of cases had bilateral involvement. However, this proportion was lower than the 76-82.6% reported in some previous studies (12, 15). This variation may be partly explained by the different composition of histological subtypes across studies.

The patients in our study were relatively young at initial BC diagnosis, with a median age of 39 years (range: 29–59 years), consistent with previous reports of BC with OM (8, 12, 14, 15). This finding suggests that younger age may be a risk factor for the development of OM, possibly related to hormonal factors or more aggressive tumor biology in younger patients. Moreover, we observed that patients with IDC were significantly younger at initial diagnosis compared to those with ILC (median 37 *vs*. 45 years), which aligns with the established epidemiology of these subtypes (28, 29, 33–35). However, it is noteworthy that even the ILC patients in our OM cohort were younger than typical ILC patients without OM (23, 35), suggesting that early-onset ILC may carry a higher risk of OM.

Our analysis of the OS from initial diagnosis revealed important insights. The median OS for the entire cohort was 85.0 months, which is slightly longer than the 72 months reported in a retrospective study that included 24 patients (12). Furthermore, our results showed that the median OS was 94.0 months for patients with IDC and 80.5 months for those with ILC, without significant differences. This suggests that while histological type may influence the disease course, the overall prognosis from initial diagnosis might be comparable between subtypes. Notably, we found that the first metastatic site significantly influenced overall prognosis. Patients with ovarian or other visceral sites as the initial metastatic site demonstrated a substantially poorer prognosis compared to those with chest wall or lymph node involvement, consistent with the established understanding that visceral metastasis suggests worse outcomes (11). This highlights that BC patients with OM often present with advanced disease burden, which contributes to their unfavorable prognosis.

One significant finding of our study is that the time from the initial diagnosis of BC to the occurrence of OM was an independent prognostic factor for OS, with a longer duration from diagnosis to OM being associated with improved OS. Specifically, the mortality risk associated with OM occurring after six years was only 15.7% of the risk associated with ovarian metastasis occurring within three years. This finding is consistent with the established oncological principle that a longer disease-free interval often reflects a more indolent tumor biology with lower metastatic potential and a less aggressive disease course (36–38). Conversely, a short interval to metastasis is a hallmark of highly aggressive and rapidly progressive disease. These findings support the conclusion that a longer interval from initial diagnosis to OM correlates with better OS outcomes. From a clinical perspective, the time-to-OM interval may serve as a valuable prognostic indicator that could help guide treatment decisions and patient counseling. Patients with longer intervals may be more likely to benefit from aggressive local and systemic therapies, while those with short intervals might be candidates for palliative approaches or clinical trials of novel agents.

Moreover, we also analyzed the OS after OM. The median OS after OM was found to be 28 months for the entire cohort, which is similar to the 25 months reported in a previous study (12) but shorter than the 49.5 months reported in another retrospective study (14). This discrepancy may be attributed to differences in cohort composition, detection methods, or treatment approaches across studies. Although the impact of surgical extent on prognosis remains debated, several studies suggest that debulking surgery, particularly when achieving minimal residual disease (< 5 mm), may improve survival outcomes of BC with OM (14, 15, 39–41). In our study, all patients underwent bilateral oophorectomy, while those with more extensive procedures (peritoneum/uterus resection) were excluded. The fact that we still identified histological type as an independent prognostic factor for OS after OM despite this standardized surgical approach strengthens the conclusion that biological differences, rather than surgical variations alone, drive the observed survival disparity. Additionally, factors such as age at the time of metastasis, unilateral or bilateral OM, and levels of CA-125 and CA-15–3 were not found to be associated with OS after OM in our cohort.

Strikingly, we observed a significant difference in post-OM survival between histological subtypes. The median OS after OM in patients with ILC was only 11 months, significantly shorter than the 30 months in patients with IDC, indicating a worse prognosis for ILC. Our data are consistent with a study involving 28 BC patients with OM, which suggests that OM is a specific negative prognostic factor for ILC (14). This profound difference in post-OM survival between histological subtypes can be attributed to several interrelated factors. First, the distinct biological behavior of ILC plays a crucial role. ILC is characterized by non-cohesive, single-file growth patterns and a pronounced propensity for diffuse peritoneal dissemination and carcinomatosis (24, 42). Moreover, it has been reported that 50% of patients with ILC have abdominal lesions, and BC patients with peritoneal metastasis generally exhibit a poor prognosis (43). This pattern of spread often remains radiologically and clinically occult until causing symptoms through extensive abdominal involvement, frequently leading to catastrophic complications such as bowel obstruction and malnutrition. Second, the clinical management for ILC further amplifies this biological disadvantage. Among the 19 cases (46.3%) whose OM were detected during ovarian ablation surgery without clinical symptoms or abnormal imaging findings—representing early, asymptomatic metastasis—18 cases were of IDC, with only 1 case of ILC. Conversely, 87.5% of ILC patients were diagnosed due to clinical symptoms, indicating advanced disease at detection. This pattern reflects fundamental differences in clinical management between the subtypes. In adjuvant settings, ovarian ablation is more frequently indicated for premenopausal women with higher-risk early-stage IDC, which more commonly presents with features like larger tumor size and higher rates of axillary lymph node metastasis (29, 35). In contrast, ILC typically presents with more favorable clinicopathological features and was historically perceived as having a better prognosis (29, 35), resulting in fewer patients receiving ovarian ablation. This management difference creates a systematic detection advantage for IDC, where OM is frequently identified incidentally at an earlier stage. The significantly longer median interval from initial BC diagnosis to OM in ILC (66 months) compared to IDC (36 months) further supports this pattern of delayed clinical presentation in ILC. Given these observations, clinicians need to maintain a high degree of suspicion for OM in patients with ILC, emphasizing the need for meticulous follow-up and possible surgical interventions despite the absence of traditional markers or symptoms.

Our study has several limitations. First, the sample size was moderate, particularly for subgroup analyses (n=8 for the ILC cohort), which may limit the statistical power of our comparisons involving this subtype, and findings related to ILC should be interpreted with caution. Future research should aim to include larger, multi-institutional cohorts to validate our findings, particularly regarding the prognostic differences between histological subtypes. Second, our cohort was restricted to patients who underwent oophorectomy, which introduces selection bias. Future research will include patients diagnosed with OM via imaging or biopsy, as well as those receiving various treatment modalities, to evaluate the prognosis of patients receiving either surgical or non-surgical systemic treatments. Third, the definition of OS after OM in our study was calculated from the date of bilateral oophorectomy to the date of death. While this approach offers valuable insights, it ties survival outcomes to a treatment event, which may obscure the true biological timing of metastatic progression. The findings for OS after OM should therefore be interpreted with an understanding that the survival interval reflects the period following pathological confirmation and surgical management. Future studies should consider alternative methodologies for establishing survival metrics, potentially incorporating time intervals from the initial detection of metastasis to enhance the accuracy and relevance of prognostic assessments.

## Conclusions

In conclusion, this study provides critical insights into the prognostic implications of OM in BC, emphasizing the notable differences between IDC and ILC. While the OS from initial diagnosis was similar between subtypes, the prognosis after the diagnosis of OM was drastically worse for patients with ILC. This disparity may be attributed to the distinct biological behavior of ILC and the later identification of OM in these patients, underscoring the necessity for heightened vigilance and potentially more aggressive surveillance within this population. Furthermore, nearly 50% of BC patients with OM presented without clinical symptoms or imaging abnormalities and were detected during ovarian ablation surgery. This suggests that early-stage OM may not manifest with overt clinical symptoms or detectable imaging findings. Additionally, the time from initial BC diagnosis to the development of OM was established as a powerful independent prognostic factor for OS, reinforcing the principle that a longer disease-free interval correlates with a more favorable outcome, which may guide clinical treatment. These findings contribute to our understanding of this challenging clinical scenario and may help guide more personalized management strategies for patients with BC and OM. Future research with larger, multi-institutional cohorts is needed to validate these findings and to guide optimal management strategies for this challenging clinical scenario.

## Data Availability

The raw data supporting the conclusions of this article will be made available by the authors, without undue reservation.
